# Supplementary figures and images for: A panel of phenotypically and genotypically diverse bioluminescent:fluorescent Trypanosoma cruzi strains as a resource for Chagas disease research
Source: PLoS Negl Trop Dis. 2024 May 31;18(5):e0012106. doi: 10.1371/journal.pntd.0012106 (PMC11168640; doi:10.1371/journal.pntd.0012106)

## Slide 1
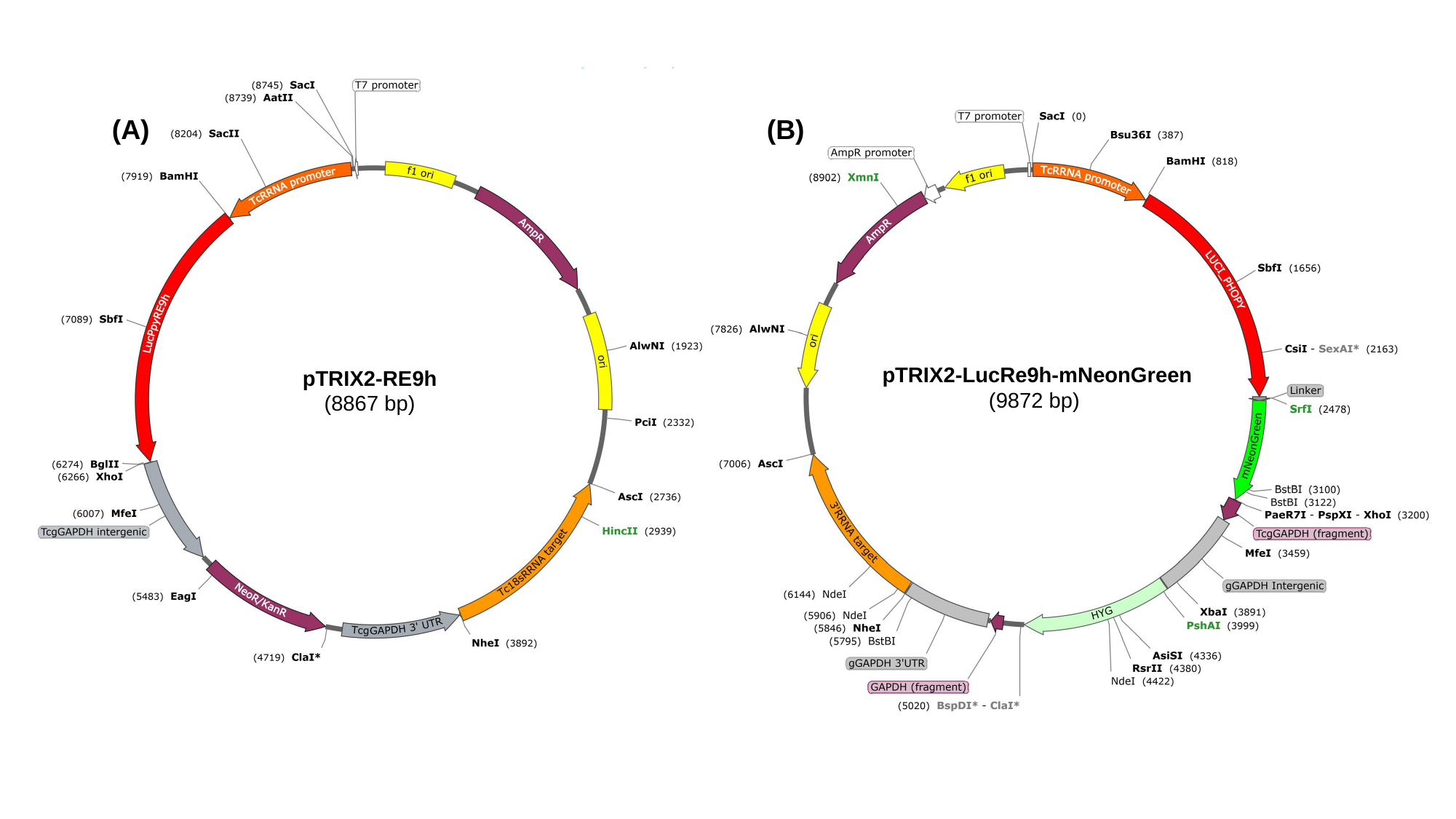

pTRIX2-RE9h
(8867 bp)
pTRIX2-LucRe9h-mNeonGreen
(9872 bp)
(A)						(B)

Supplement: S1 Fig — (A) The red-shifted luciferase gene PpyRE9h [31] can be targeted to T. cruzi rDNA loci after transfection with a 6 kb fragment produced by AatII/AscI digestion of construct pTRIX2-RE9h and selection with G418 [13,30]. (B) The PpyRE9h:mNeonGreen fusion sequence [17] can be similarly targeted following transfection with a 7.0 kb SacI/AscI fragment fragment from construct pTRIX2-LucRe9h-mNeonGreen, and selection with hygromycin. Construct sequences are available on GenBank (Materials and Methods). The images were generated using SnapGene software (www.snapgene.com) (PPTX) [file pntd.0012106.s001.pptx]

## Slide 1
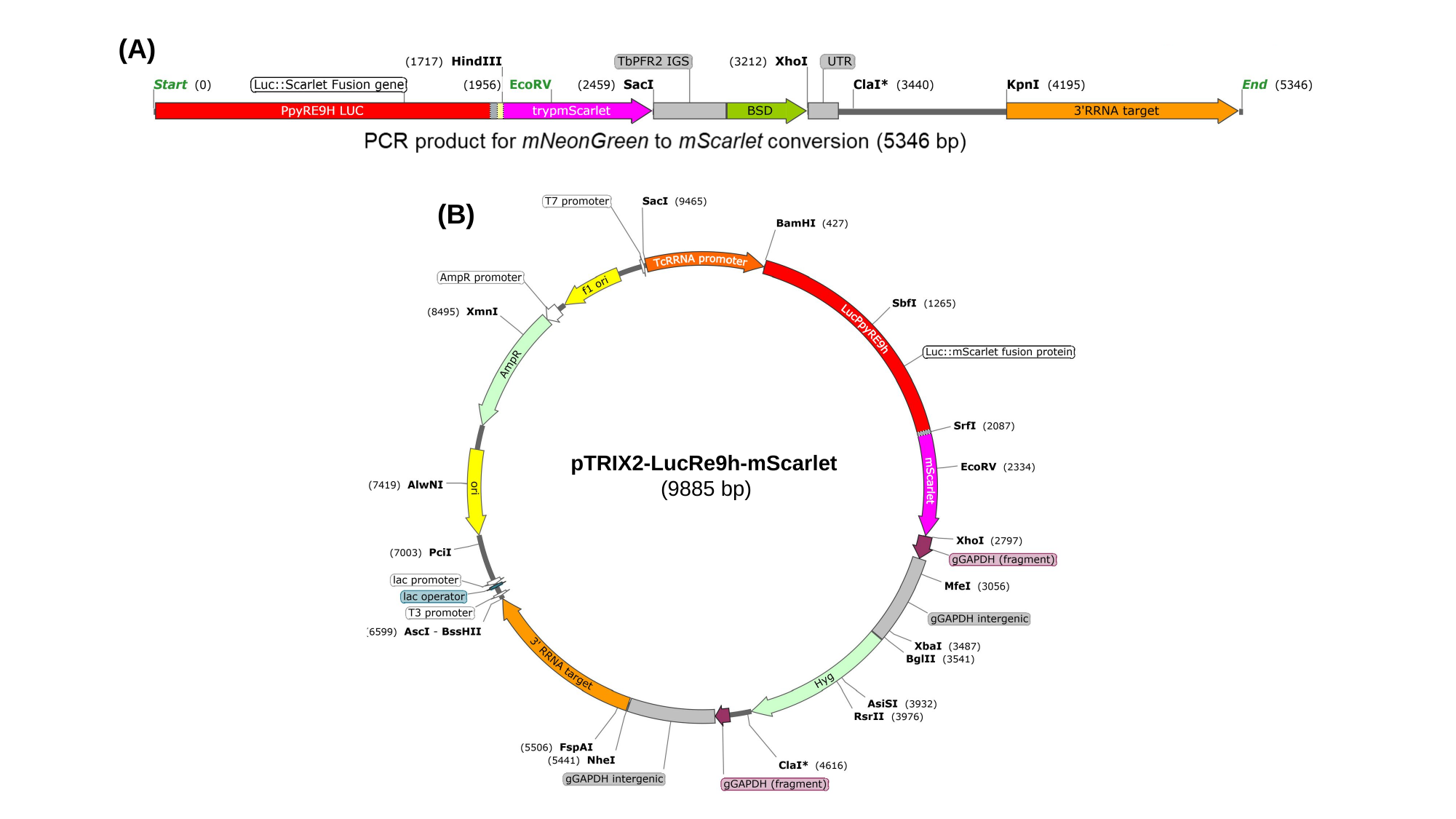

(A)
pTRIX2-LucRe9h-mScarlet
 (9885 bp)
(B)

Supplement: S2 Fig — (A) Map of the 5.35 kb PCR fragment used to convert the fluorescent component of the dual reporter protein from mNeonGreen to mScarlet with the blasticidin S deaminase gene (BSD) as the selectable marker [17]. (B) Construct pTRIX2-LucRe9h-mScarlet which contains a 7.0 kb SacI/AscI fragment that can be used to transfect T. cruzi and generate red fluorescent variants following selection with hygromycin. Sequences of fragment and construct are available on GenBank (Materials and Methods). The images were generated using SnapGene software (www.snapgene.com). (PPTX) [file pntd.0012106.s002.pptx]

## Slide 1
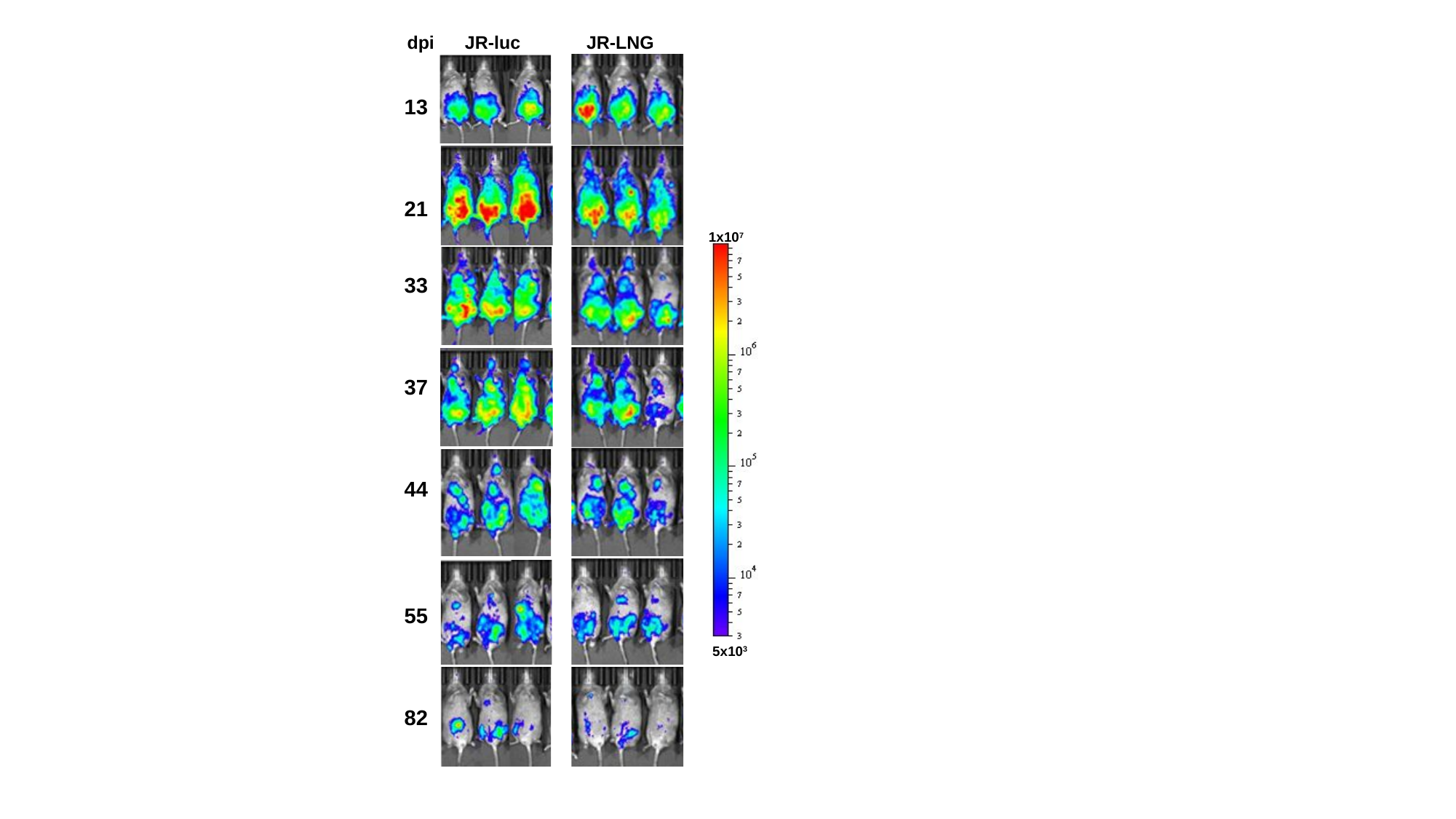

dpi JR-luc JR-LNG
13
21
33
37
44
55
82
1x107
5x103

Supplement: S3 Fig — C3H/HeN mice were injected i.p. with 5 x 10^4^ trypomastigotes expressing either the red-shifted luciferase (JR-Luc) or the luciferase:mNeonGreen fusion protein (JR-LNG) (Materials and Methods). They were monitored by in vivo imaging, at the days indicated post-infection (dpi). The heat-map indicates the intensity of bioluminescence. (PPTX) [file pntd.0012106.s003.pptx]

## Slide 1
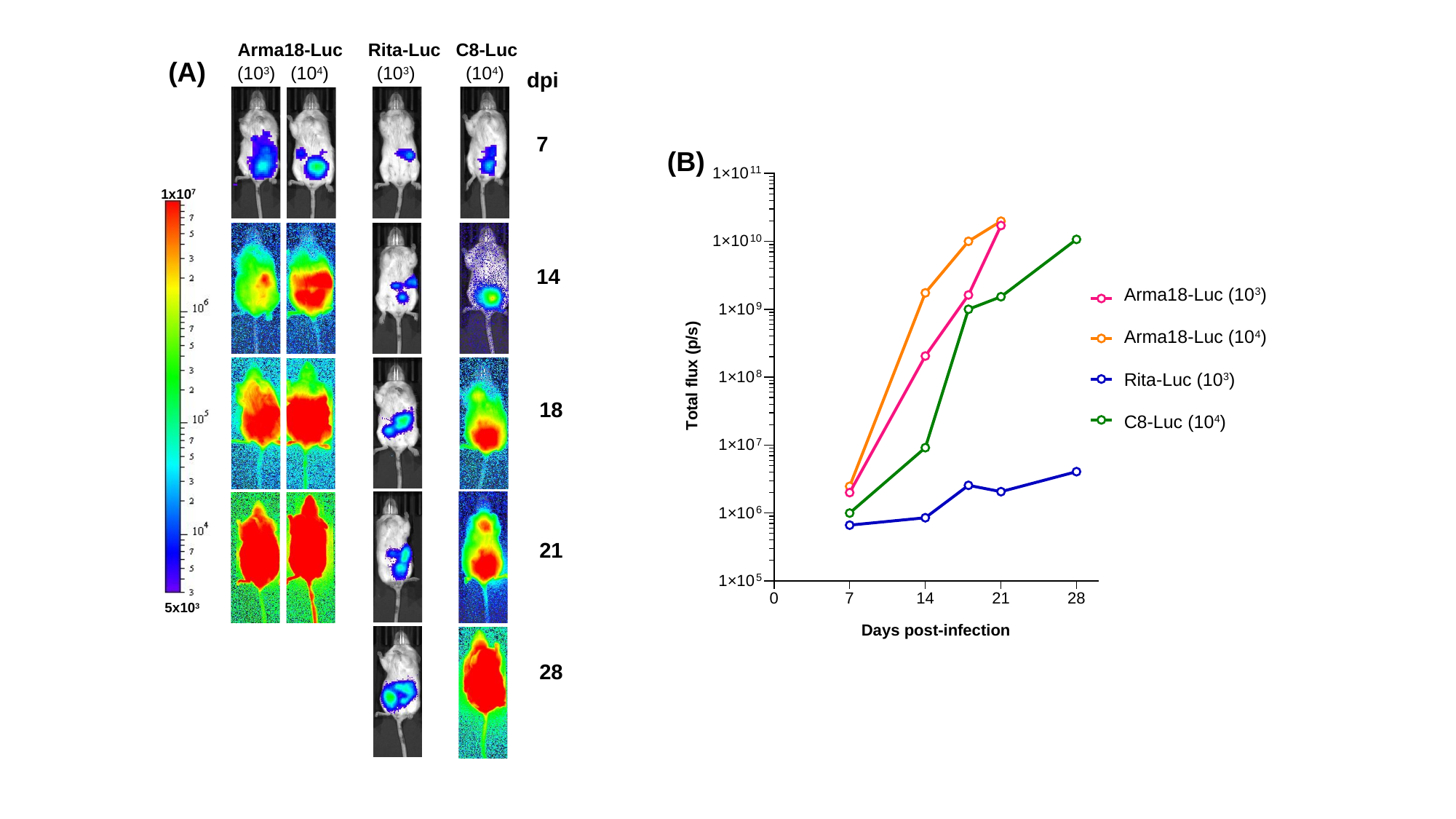

Arma18-Luc Rita-Luc C8-Luc
(A)
(103) (104)	 (103) (104)
dpi
7
(B)
1x107
5x103
14
Arma18-Luc (103)
Arma18-Luc (104)
Rita-Luc (103)
C8-Luc (104)
18
21
28

Supplement: S4 Fig — Mice were infected i.p. with bioluminescent tissue culture trypomastigotes (numbers shown in brackets) and monitored by in vivo imaging (Materials and Methods) at the days indicated post-infection (dpi). The heat-map indicates the intensity of bioluminescence. (B) Total bioluminescence flux derived by ventral imaging at the days indicated. (PPTX) [file pntd.0012106.s004.pptx]

## Slide 1
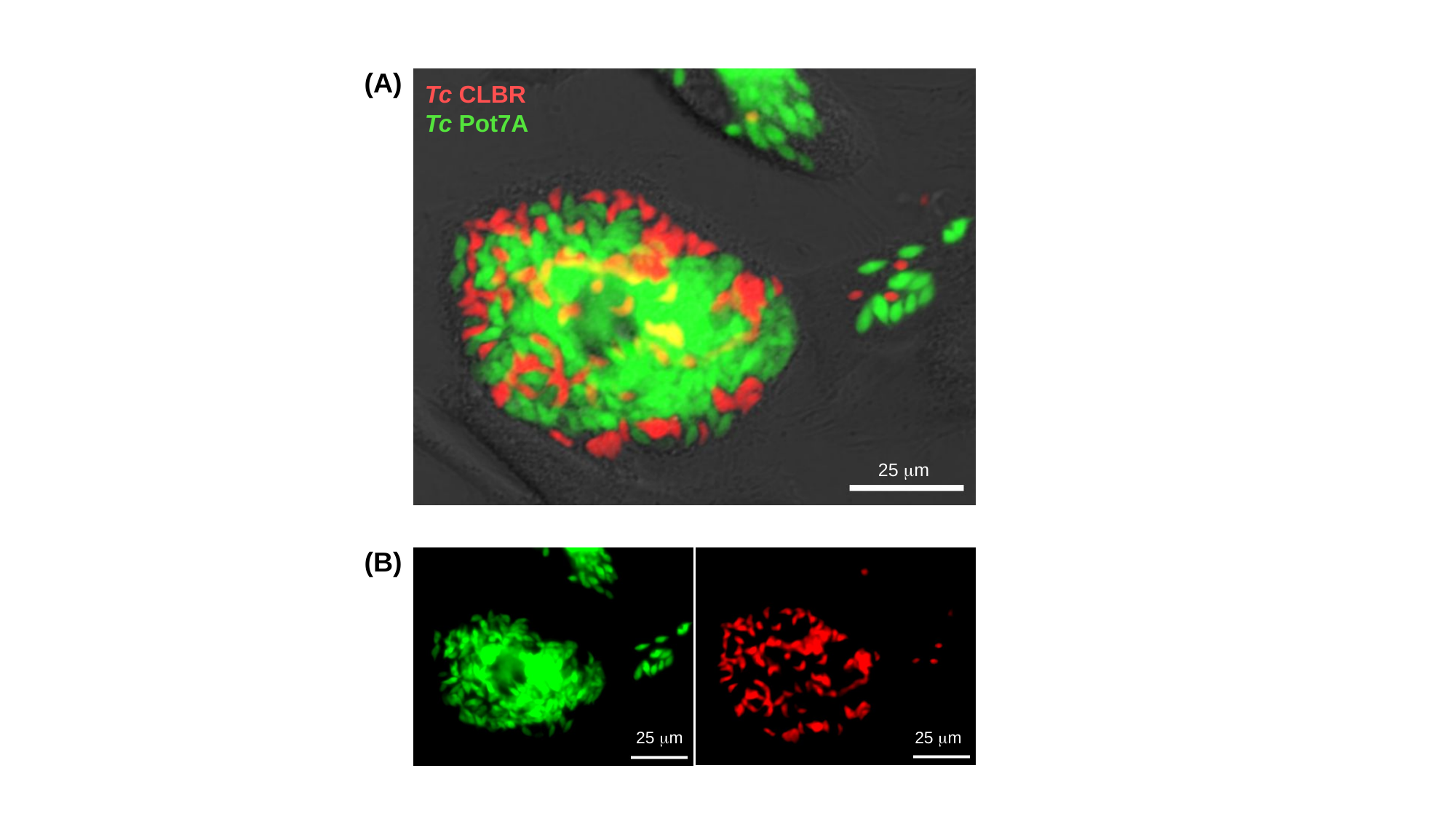

(A)
Tc CLBR
Tc Pot7A
25 mm
(B)
25 mm
25 mm

Supplement: S5 Fig — Human foreskin fibroblast (HFF) cells were infected with T. cruzi Pot7a-Luc:NeonGreen (Tc Pot7a –green) and 5 days later with T. cruzi CLBR-Luc:mScarlet (Tc CLBR–red). (A) Image taken with a Nikon Ti-2 E inverted microscope using red and green filters, 5 days after the second infection. (B) Same image captured with red or green filters. Epifluorescence real-time microscopy can also be used to track co-infections. (PPTX) [file pntd.0012106.s005.pptx]
